# Supplementary material for: Steering and cloaking of hyperbolic polaritons at deep-subwavelength scales
Source: Nat Commun. 2024 May 25;15:4463. doi: 10.1038/s41467-024-48318-w (PMC11127984; doi:10.1038/s41467-024-48318-w)
Supplement: Supplementary file 1 — Supplementary Information [file 41467_2024_48318_MOESM1_ESM.pdf]

Supplementary Information for

Steering and cloaking of hyperbolic polaritons at deep-subwavelength scales

Hanchao Teng,[1,2†] Na Chen,[1,2†] Hai Hu,[1,2*] F. Javier García de Abajo,[3,4] D. N. Basov,[5] Qing Dai[1,2*]

1 CAS Key Laboratory of Nanophotonic Materials and Devices, CAS Key Laboratory of Standardization and Measurement for Nanotechnology, CAS Center for Excellence in Nanoscience, National Center for Nanoscience and Technology, Beijing 100190, P. R. China.
2 Center of Materials Science and Optoelectronics Engineering, University of Chinese Academy of Sciences, Beijing 100049, P. R. China.
3 ICFO-Institut de Ciencies Fotoniques, The Barcelona Institute of Science and Technology, 08860 Castelldefels (Barcelona), Spain.
4 ICREA-Institució Catalana de Recerca i Estudis Avançats, Passeig Lluís Companys 23, 08010 Barcelona, Spain.
5 Department of Physics, Columbia University, New York 10027, United States.

* e-mail: daiq@nanoctr.cn, huh@nanoctr.cn

† These authors contributed equally

This PDF file includes:

Supplementary Note 1. Discussion of fringe formation.

Supplementary Figure 1. Illustration of different paths from which the signals can be collected by the detectors.

Supplementary Figure 2. Extraction of antenna-launched hybrid polaritons.

Supplementary Figure 3. Illustration of the device structure for theoretical modeling.

Supplementary Note 2. Calculation of dispersion and isofrequency contours (IFCs) of twisted phonon polaritons.

Supplementary Figure 4. Experimental and theoretically calculated spatial Fourier transforms and IFCs.

Supplementary Figure 5. Deflection angle and full width at half maximum (FWHM) for refraction of hyperbolic polaritons.

Supplementary Figure 6. Analysis of refractive transmission from hyperbolic polaritons to topological polaritons in twisted structures.

Supplementary Note 3. Calculation of transmittance and polariton quality factors.

Supplementary Note 4. Real-space simulation of hybrid polaritons in twisted α-MoO_3 films.

Supplementary Figure 7. Numerically simulated refractive transmission of polaritons as they reach the twisted region with different twisting angles.

Supplementary Figure 8. Numerically simulated polariton transmittance across the interface for different thicknesses of the top α-MoO_3 layer.

Supplementary Figure 9. Numerically simulated near-field distribution of polariton refraction.

Supplementary Figure 10. Illustration of the fabrication process.

Supplementary Figure 11. Three-dimensional topography images and height profiles of the in-plane jigsaw structures.

Supplementary Figure 12. Control experiments for an in-plane jigsaw to steer polaritons.

Supplementary Figure 13. Numerically simulated near-field distribution of polariton refraction controlled by the top tailored α-MoO_3 micro-ribbons.

Supplementary Figure 14. Comparison of near-field images of the cloaking device before and after introducing a defect.

Supplementary Figure 15. Control experiments for comparison of polariton propagation with and without introducing a defect when there is no cloaking structure.

Supplementary References

Supplementary Note 1. Discussion of fringe formation

The signals observed in near-field imaging can be categorized into five pathways of light in the measurement, as depicted in Supplementary Figure 1. These paths are classified as P_1 to P_5. P_1 denotes the fraction of incident light that is directly reflected by the tip (black, tip-reflected). P_2 shows the outward propagation of polaritons launched by the tip, which are subsequently scattered into the detector by the sample edge (blue, tip-launched/edge-scattered). P_3 involves tip-launched polaritons that propagate outward to the edge, reflect, and then scatter into the detector after interacting with the tip (orange, tip-launched/edge-reflected/tip-scattered). P_4 illustrates polaritons launched from the edge and scattered into the detector by the tip (green, edge-launched/tip-scattered). Lastly, P_5 demonstrates resonant antenna-launched polaritons that are scattered into the detector by the tip (red, antenna-launched/tip-scattered). More details about the discussion and analysis of fringe formation have been reported in several previous studies[1, 2].

In s-SNOM measurements, various structures such as tips, antennas, edges, and even defects can serve as exciters for polaritons. Although we primarily utilize metal antennas as the excitation source, one can expect that the antenna-launched hyperbolic wave should be surrounded by edge-parallel fringes from both tip- and edge-launched polaritons, as illustrated in Supplementary Figure 2a. To eliminate the disturbance of fringes, we extract antenna-launched phonon polaritons from the complex background signals (Supplementary Figure 2b) by following a filtering method described in previous studies[3, 4].

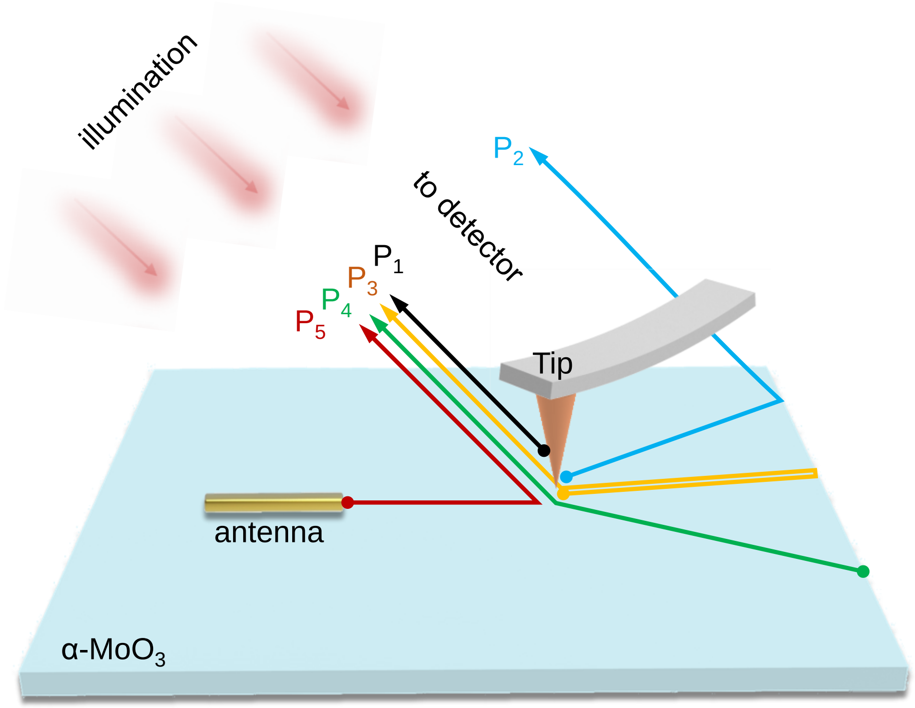

Supplementary Figure 1. Illustration of different paths from which the signals can be collected by the detectors. P_1 to P_5 represent different signal pathways collected by the s-SNOM detectors.

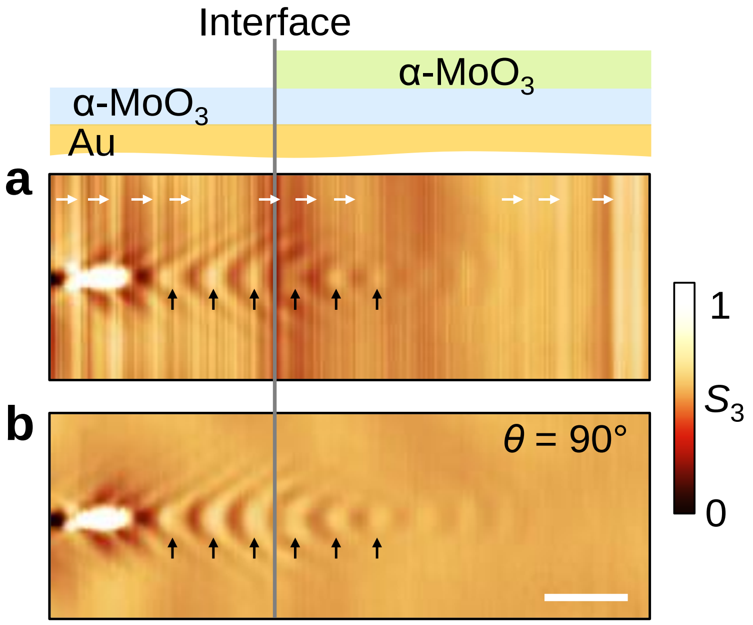

Supplementary Figure 2. Extraction of antenna-launched hybrid polaritons. (**a**) Raw data of the near-field image, as recorded by our s-SNOM. (**b**) Near-field image of antenna-launched hybrid polaritons corresponding to the raw data in (a), obtained by subtracting the vertical polariton fringes launched or reflected by the edges. The scale bar indicates 3 μm. The collected near-field amplitude is the combination of antenna-launched polaritons and the local scattering amplitude resulting from the dielectric response of the material beneath the tip. An abrupt jump in amplitude would be expected at the interface between the single- and double-layer regions. For very thin samples, such as graphene on *h*-BN,[5] the intensity signal indeed varies as a function of the number of layers. However, this abrupt jump in amplitude is not found in our experiments. We attribute this observation to the thicker thickness of our α-MoO_3 film and the use of a gold substrate. In our structure, the thickness of the bottom α-MoO_3 is quite substantial, ranging from approximately 300 nm to 550 nm. Consequently, the local scattering amplitude detected by the tip predominantly originates from the material itself. In addition, when dealing with general dielectric substrates like SiO_2, variations in material thickness can lead to changes in the local scattering amplitude, particularly when the thickness is less than half a wavelength. However, the evanescent field is unable to penetrate the metal in our experiments, resulting in total reflection occurring on the gold substrate. This explanation aligns with findings from various previous independent studies.[6-8]

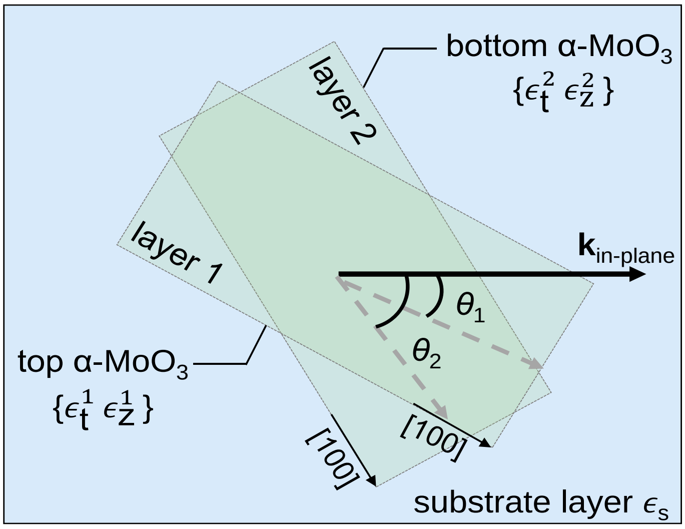

Supplementary Figure 3. Illustration of the device structure for theoretical modeling. Top view of stacking of a twisted α-MoO$_3$ heterostructure. Layers 1, 2, and 3 correspond to the top α-MoO$_3$ film, bottom α-MoO$_3$ film, and substrate layer, respectively. Two α-MoO$_3$ layers are described by their dielectric tensor $(\epsilon_t^1, \epsilon_z^1)$, $(\epsilon_t^2, \epsilon_z^2)$ and thickness d_1, d_2, respectively. The angles θ_1 and θ_2 indicate the [100] crystal orientations of the two layers relative to the optical in-plane wave vector $\mathbf{k}_{\text{in-plane}}$.

Supplementary Note 2. Calculation of dispersion and isofrequency contours (IFCs) of twisted phonon polaritons

In the theoretical model, we consider a twisted α-MoO_3 heterostructure as a stratified medium of infinite lateral extension (Supplementary Figure 3). The top layer (layer 0) and the bottom layer (layer 3) represent the surrounding air and Au substrate, respectively, characterized by their respective dielectric functions ϵ_a and ϵ_s (ref. 35 in the main text). We consider two layers of α-MoO_3 that have a twisting angle θ. Each layer can be modeled as a biaxial anisotropic material with a diagonal dielectric function tensor $\{\epsilon_x, \epsilon_y, \epsilon_z\}$ along principal orthogonal axes (ref. 16 in the main text). Assuming wave propagation along the $\mathbf{k}_{\text{in-plane}}$ direction, the dielectric function of each layer can be described as $\epsilon_t = \epsilon_x \cos^2\theta_j + \epsilon_y \sin^2\theta_j$, where θ_j is the angle between the $\mathbf{k}_{\text{in-plane}}$ and crystal orientation [100] of α-MoO_3 in that layer, as shown in Supplementary Figure 3.

By solving Maxwell's equations, we can obtain the $E_x(z)$ and $H_y(z)$ fields of each α-MoO_3 layer (all sharing a global $e^{i\mathbf{k}_{\text{in-plane}}\cdot(x,y)}$ in-plane dependence),

$$H_y(z) = \begin{cases} \left(Q(1)+Q(2)\right)e^{-\alpha_a z} & (z>0) \\ Q(1)e^{ik_z^{(1)}z} + Q(2)e^{-ik_z^{(1)}z} & (-d_1 < z \le 0) \\ Q(3)e^{ik_z^{(2)}z} + Q(4)e^{-ik_z^{(2)}z} & (-d_2 < z \le -d_1) \\ \left(Q(3)e^{-ik_z^{(2)}d_2} + Q(4)e^{ik_z^{(2)}d_2}\right)e^{\alpha_s(z+d_2)} & (z \le -d_2) \end{cases} \quad (S1)$$

$$E_x(z) = \begin{cases} \frac{i\alpha_a}{\omega\epsilon_0\epsilon_a}\left(Q(1)+Q(2)\right)e^{-\alpha_a z} & (z>0) \\ \frac{1}{\omega\epsilon_0\epsilon_t^{(1)}}\left(Q(1)k_z^{(1)}e^{ik_z^{(1)}z} - Q(2)k_z^{(1)}e^{-ik_z^{(1)}z}\right) & (-d_1 < z \le 0) \\ \frac{1}{\omega\epsilon_0\epsilon_t^{(2)}}\left(Q(3)k_z^{(2)}e^{ik_z^{(2)}z} - Q(4)k_z^{(2)}e^{-ik_z^{(2)}z}\right) & (-d_2 < z \le -d_1) \\ \frac{-i\alpha_s}{\omega\epsilon_0\epsilon_s}\left(Q(3)e^{-ik_z^{(2)}d_2} + Q(4)e^{ik_z^{(2)}d_2}\right)e^{\alpha_s(z+d_2)} & (z \le -d_2) \end{cases} \quad (S2)$$

where $\alpha_{a,s} = \sqrt{\left(q^2 - k_0^2\epsilon_{a,s}\right)}$ represents the evanescent attenuation coefficient of air (layer 0) and the substrate (layer 3); $k_z^{(j)} = \sqrt{k_0^2\epsilon_t^{(j)} - \left(\frac{\epsilon_t^{(j)}}{\epsilon_z^{(j)}}\right)q^2}$ indicates the vertical component of the wave vector in the α-MoO_3 layer $j = 1,2$; we define $q = \left|\mathbf{k}_{\text{in-plane}}\right|$; the amplitude coefficients $Q(i)$ (i = 1, 2) represent nonzero solutions that can be obtained by imposing the electromagnetic boundary conditions (Eq. $(S3)$ below); and d_j is the thickness of layer j.

By imposing the continuity of the tangential components of the electric and

magnetic fields, $\vec{E}$ and $\overrightarrow{H}$, at the layer boundaries, we obtain the expression

$$E_x^{(j)} = E_x^{(j+1)}, H_y^{(j)} = H_y^{(j+1)} \quad \text{at} \quad z = -d_j \ (j = 0,1,2), \tag{S3}$$

where $d_0 = 0$ indicates the boundary between air and the top α-MoO_3 layer.

After substituting the fields in Eq. ($S1$) and Eq. ($S2$) into the boundary conditions (Eq. ($S3$)), we obtain the matrix equation

$$\begin{bmatrix} \frac{i\alpha_a}{\epsilon_a} - \frac{k_z^{(1)}}{\epsilon_t^{(1)}} & \frac{i\alpha_a}{\epsilon_a} + \frac{k_z^{(1)}}{\epsilon_t^{(1)}} & 0 & 0 \\ \frac{k_z^{(1)}}{\epsilon_t^{(1)}} e^{-ik_z^{(1)} d_1} & -\frac{k_z^{(1)}}{\epsilon_t^{(1)}} e^{ik_z^{(1)} d_1} & -\frac{k_z^{(2)}}{\epsilon_t^{(2)}} e^{-ik_z^{(2)} d_1} & \frac{k_z^{(2)}}{\epsilon_t^{(2)}} e^{ik_z^{(2)} d_1} \\ e^{-ik_z^{(1)} d_1} & e^{ik_z^{(1)} d_1} & -e^{-ik_z^{(2)} d_1} & e^{ik_z^{(2)} d_1} \\ 0 & 0 & \left(\frac{i\alpha_s}{\epsilon_s} + \frac{k_z^{(2)}}{\epsilon_t^{(2)}}\right) e^{-ik_z^{(2)} d_2} & \left(\frac{i\alpha_s}{\epsilon_s} - \frac{k_z^{(2)}}{\epsilon_t^{(2)}}\right) e^{ik_z^{(2)} d_2} \end{bmatrix} \begin{bmatrix} Q(1) \\ Q(2) \\ Q(3) \\ Q(4) \end{bmatrix}, \tag{S4}$$

This equation has the form $A \cdot Q = 0$, where A is a square matrix of order 4 and Q is a vector comprising the field coefficients. A nontrivial solution requires the vanishing of the determinant of A, from which we obtain the dispersion of polaritons in the twisted α-MoO_3 heterostructure[7-10], as illustrated in Supplementary Figure 4.

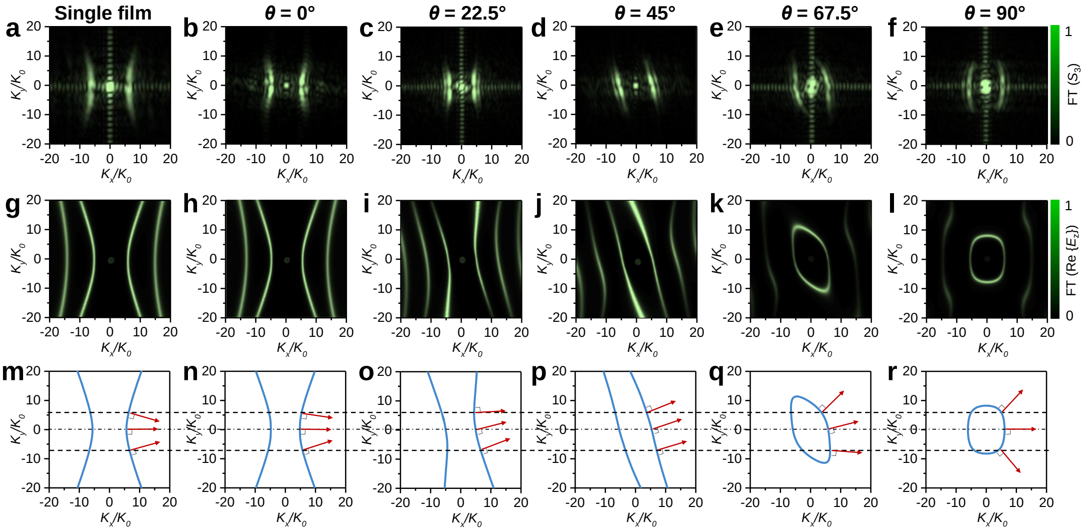

Supplementary Figure 4. Experimental and theoretically calculated spatial Fourier transforms and IFCs. (a-f) Absolute value of the spatial Fourier transforms of the experimentally acquired near-field images in Figure 1 of the main text. **(g-l)** Calculated spatial Fourier transforms. (**m-r**) IFCs of the twisted α-MoO_3 heterostructure for different twist angles, illustrating a transition from normal to negative refraction.

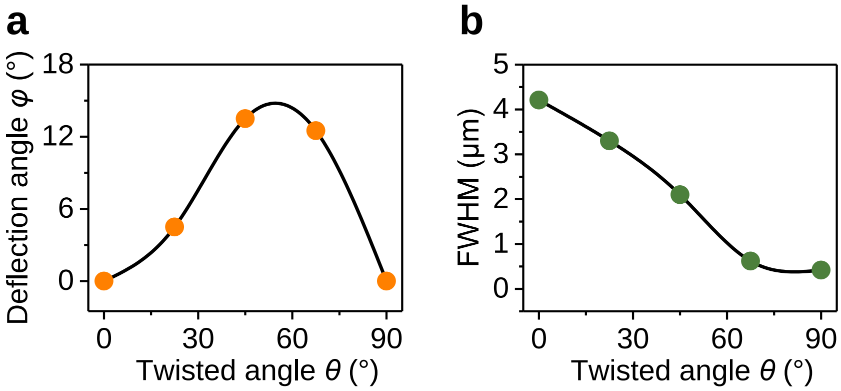

Supplementary Figure 5. Deflection angle and full width at half maximum (FWHM) for refraction of hyperbolic polaritons. (a) Experimentally measured in-plane deflection angle φ of refracted polaritons as a function of twist angles in Figures 1b-f of the main text. **(b)** FWHM of the 4th fringes of polaritons in Figures 1b-f of the main text. Black-solid curves are guides to the eye.

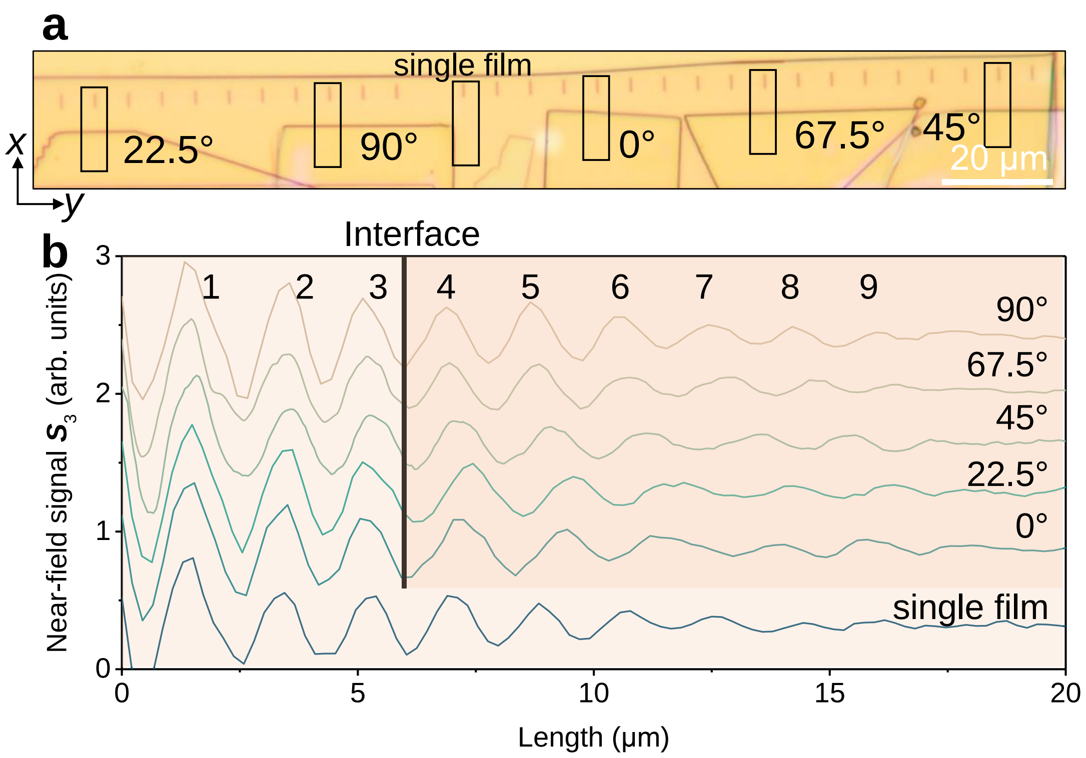

Supplementary Figure 6. Analysis of refractive transmission from hyperbolic polaritons to topological polaritons in twisted structures. (a) Optical image of a single α-MoO_3 film covered by tailored α-MoO_3 films at different twist angles θ = 0°, 22.5°, 45°, 67.5°, and 90°, corresponding to the sample in Figure 1 of the main text. The scale bar indicates 20 μm. **(b)** Near-field amplitude profiles along the polariton propagation path in Figures 1b-f of the main text.

Supplementary Note 3. Calculation of transmittance and polariton quality factors

As a result of the difference in polariton wavelength on the two sides of the interface, obtaining an accurate quality factor for the entire polariton (i.e., including incoming, transmitted, and reflected components), which combine hyperbolic polaritons at the near side of the interface with hybrid polaritons at the far side, is challenging using a conventional definition of this quantity. We thus define an *ad hoc* overall quality factor Q by comparing the intensities of the refractively propagated polaritons with those of polaritons in a bare version of the bottom layer. This involves taking the average of the intensity ratios of each corresponding fringe. More precisely,

$$Q = Q_s \frac{1}{8} \sum_{n=2}^{9} \frac{I_{an}/I_{a1}}{I_{sn}/I_{s1}}, \qquad (S5)$$

where I denotes the near-field amplitude of the fringes along the propagation direction, while the subscripts a and s represent the twisted structure and the bottom single slab, respectively. The subscript n runs over the order of the fringes, as shown in Supplementary Figure 6b. We include an overall factor $Q_s = 30$ representing a fitting result of hyperbolic polaritons in the bottom single film.

The transmittance at the interface can also be determined by comparing the intensity ratio of fringes on both sides of the interface to that of the single film according to the expression

$$T = \left(\frac{I_{a4}/I_{a3}}{I_{s4}/I_{s3}}\right)^2. \qquad (S6)$$

Supplementary Note 4. Real-space simulation of hybrid polaritons in twisted α-MoO_3 films

A finite-element method was used to simulate polaritons in twisted α-MoO_3 layers in real space using the commercial software COMSOL Multiphysics. A plane wave polarized along the long axis of the antenna at an angle of 45º relative to the surface was used to launch polaritons. Perfectly matched layers were introduced to reduce boundary reflections. At 20 nm above the uppermost surface of the sample, the electric field distribution $\mathrm{Re}\{E_z\}$ was recorded. The permittivity of the gold substrate and antennas was obtained from ref. 35 in the main text.

In the simulations presented in Supplementary Figures 7 and 8, polaritons were launched from a port to the left and propagated through the nonuniform double films with an increasing number of layers toward the right port.

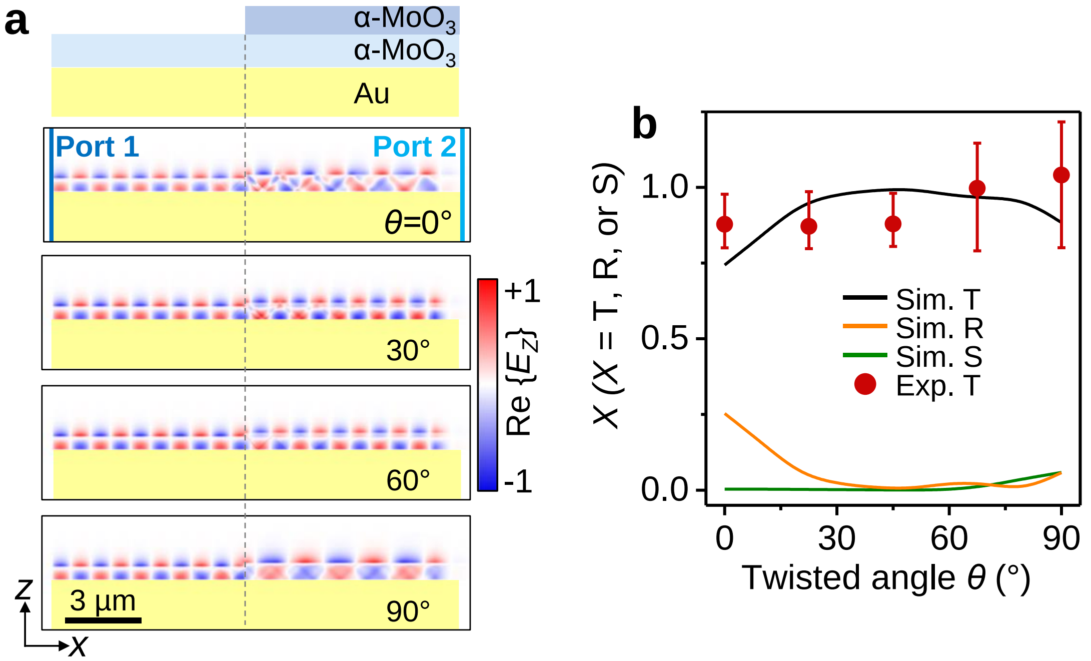

Supplementary Figure 7. Numerically simulated refractive transmission of polaritons as they reach the twisted region with different twisting angles. (**a**) Numerically simulated spatial distributions (Re{E_z}) of polaritons transmission across the interface, illustrating mode matching of field profiles for different twisting angles. Polaritons were launched from a port to the left and then propagated in the bottom α-MoO_3 film to the twisted region toward a port to the right. (**b**) Numerically simulated transmittance (black), reflectance (orange), and scattering (green) as a function of twisted angle *θ*. The red dots correspond to the experimental collection in Supplementary Note 2. The transmittance T (reflectance R, scattering S) at the interface was defined as the ratio of the transmitted (reflected, scattered) polariton energy to that of incident polaritons. The parameters of materials and frequencies are consistent with the experiments in Figure 1 of the main text. Beyond a twisted angle of 60°, experimental measurements reveal a higher transmittance when compared to simulated outcomes. This disparity can be attributed to the phenomenon of negative refraction engendered by the polaritons upon traversing the interface, thereby yielding an intensified focusing effect that, in turn, leads to a heightened electric field distribution in the experiments.

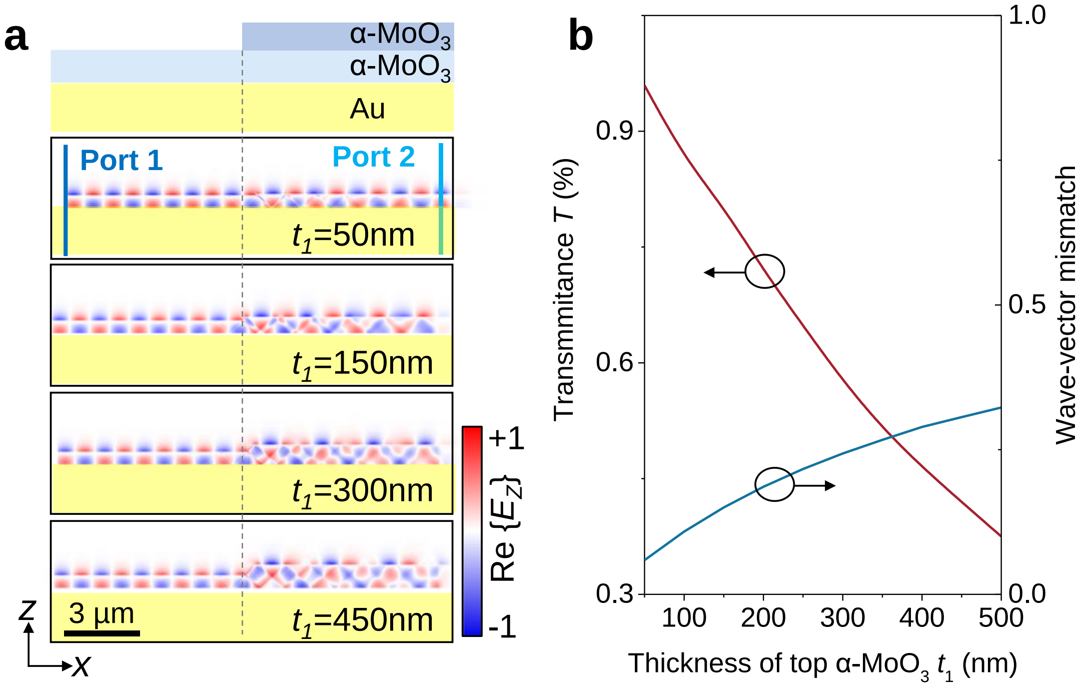

Supplementary Figure 8. Numerically simulated polariton transmittance across the interface for different thicknesses of the top α-MoO_3 layer. (**a**) Simulated near-field (Re{E_Z}) image illustrating the refractive transmission of polaritons for different thicknesses of the top α-MoO_3 film. (**b**) Numerically simulated transmittance (red) and wave vector mismatch (blue) as a function of the thickness of the top α-MoO_3 layer.

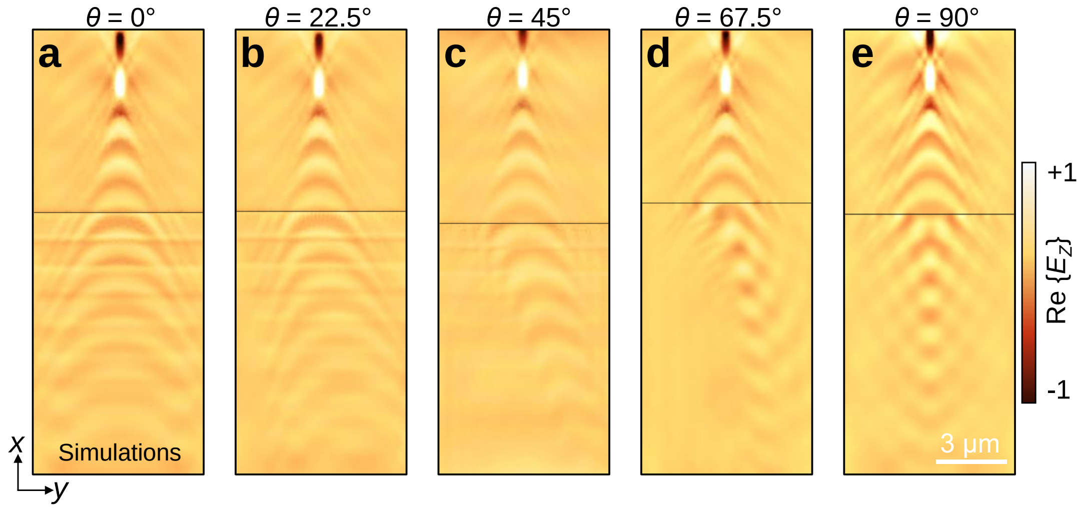

Supplementary Figure 9. Numerically simulated near-field distribution of polariton refraction. We present results corresponding to the experimental measurements in Figures 1b-f of the main text. The illumination frequency is 893 cm^{-1} and the scale bar indicates 3 μm.

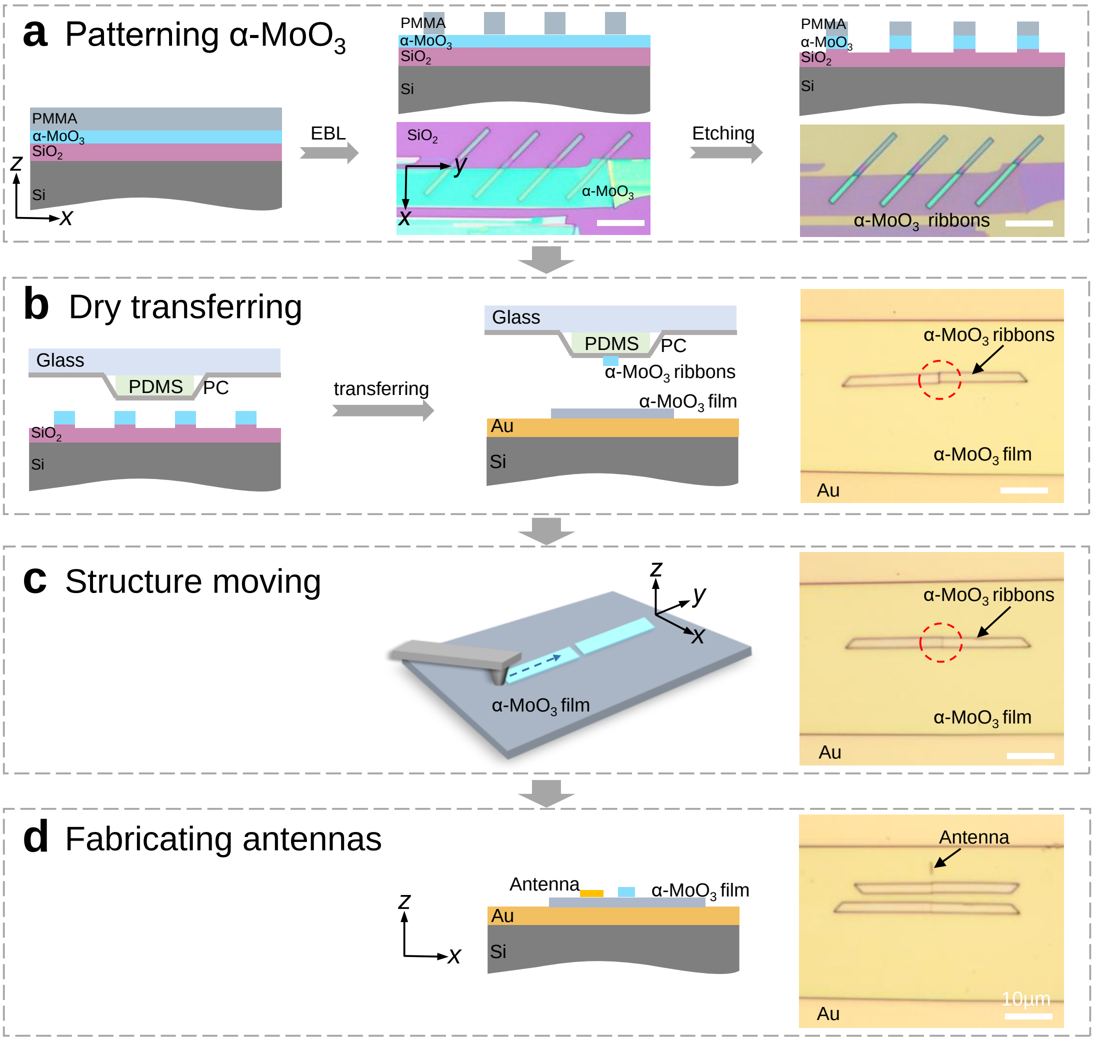

Supplementary Figure 10. Illustration of the fabrication process. (**a**) **Patterning α-MoO_3.** Oriented edges of the microribbons were patterned on a selected α-MoO_3 film on 300 nm SiO_2/500 μm Si substrate using 100 kV electron-beam lithography (EBL) (Vistec 5000+ES, Germany) with approximately 1 μm of PMMA resist. The patterns were then etched with SF_6 and Ar. (**b**) **Dry transfer.** The as-prepared α-MoO_3 ribbons were picked up from the SiO_2/Si substrate and then spliced onto an α-MoO_3 film step by step (i.e., one at a time). (**c**) **Structure moving.** A plateau AFM tip was used to act as a *bulldozer* and push or rotate structures to specific positions. (**d**) **Antenna fabrication.** Gold antennas were deposited using electron-beam evaporation in a vacuum chamber at a pressure of <5×10^{-6} Torr, followed by liftoff to remove any residual organic materials and Au. Scale bars indicate 10 μm in all panels.

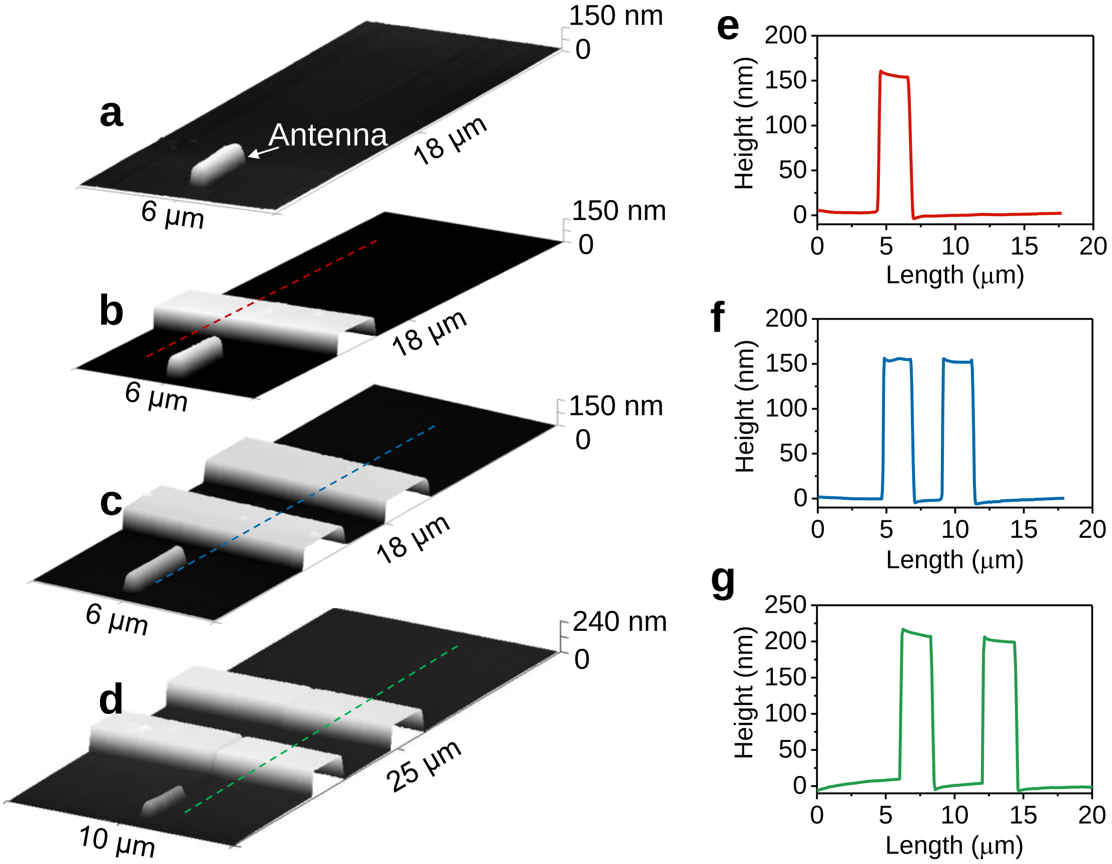

Supplementary Figure 11. Three-dimensional topography images (a-d) and height profiles (e-g) of the in-plane jigsaw structures. We present results corresponding to the samples in Figure 2 and Figure 3 of the main text.

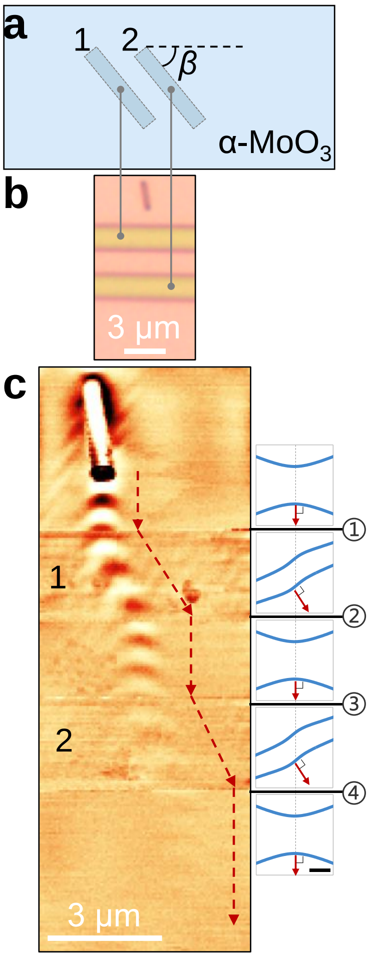

Supplementary Figure 12. Control experiments for an in-plane jigsaw to steer polaritons. (a) Illustration of tailoring α-MoO_3 microribbons with cut-edge angles *β*. (**b**) Optical images of the polariton device composed of a bottom α-MoO_3 film and tailored α-MoO_3 microribbons. Two cut α-MoO_3 microribbons with the same edge orientation *β*=45° (labeled 1 and 2) are used. (**c**) Near-field amplitude images of the polaritonic device correspond to the optical image in panel (b). The polariton propagation path is dominated by tailored α-MoO_3 microribbons, forming a sequence of two shifts to the right. Red-dashed arrows indicate the main path of the polaritonic propagation, corresponding to the red arrows in the IFCs on the right part of each panel. The illumination frequency is fixed at 900 cm^{-1}. The thicknesses of the bottom film and top ribbons are 184 nm and 154 nm, respectively. Scale bars indicate 3 μm in real-space images and 20 k_0 in dispersion diagrams.

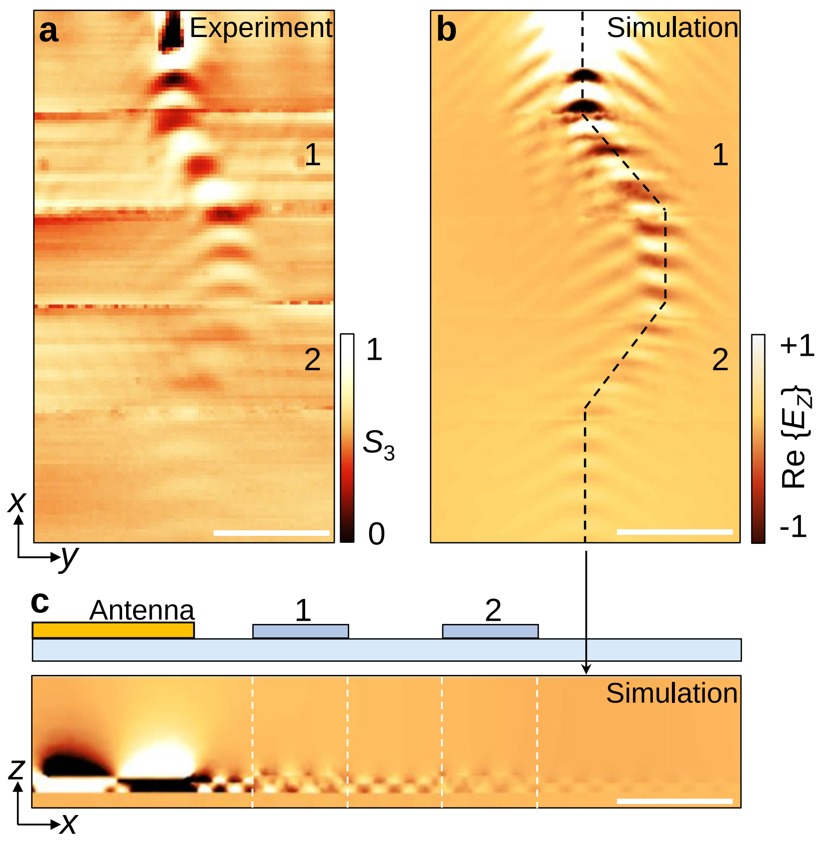

Supplementary Figure 13. Numerically simulated near-field distribution of polariton refraction controlled by the top tailored α-MoO_3 micro-ribbons. (**a**) The same experimental image of Figure 2l in the main text. (**b**) Simulated near-field (Re{E_z}) image illustrating the polariton refractions controlled by the top tailored α-MoO_3 microribbons. (**c**) The extracted distribution of the out-of-plane electric field (Re{E_z}) along the black-dashed lines in panel (b). Scale bars in all panels indicate 3 μm.

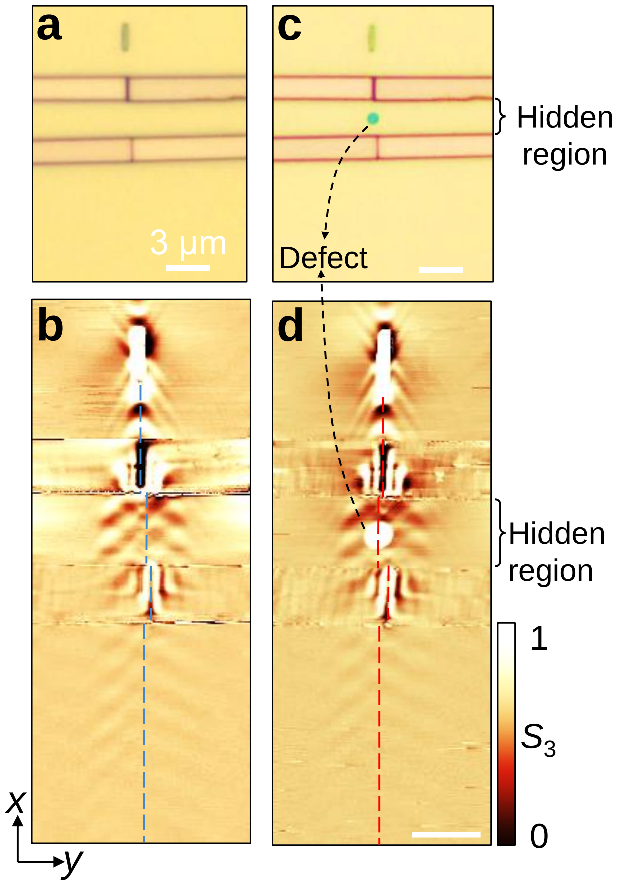

Supplementary Figure 14. Comparison of near-field images of the cloaking device before and after introducing a defect. (**a, c**) Optical images for the *in situ* device with (c) and without (a) a defect. (**b, d**) Experimentally acquired near-field amplitude images corresponding to the devices in panels (a, c), respectively. The scale bar indicates 3 μm. Due to the slight unintended misalignment between the microribbons, the near-field profile is extracted along four segments, which are represented by blue and red vertical dashed lines in panels (b, d). In regions 2-3 and 1-4, there are some vertically oriented fringes near the vertical edge of each ribbon. This observation is somewhat different from that in the simulations presented in the main text. We identify that these fringes originate in tip and edge excitation. As we collect images through tip-scan mapping, this is an unavoidable interference. However, in the simulation, we directly extract the field distribution, therefore eliminating such interference issues and allowing us to observe the refraction of polaritons excited by the antenna.

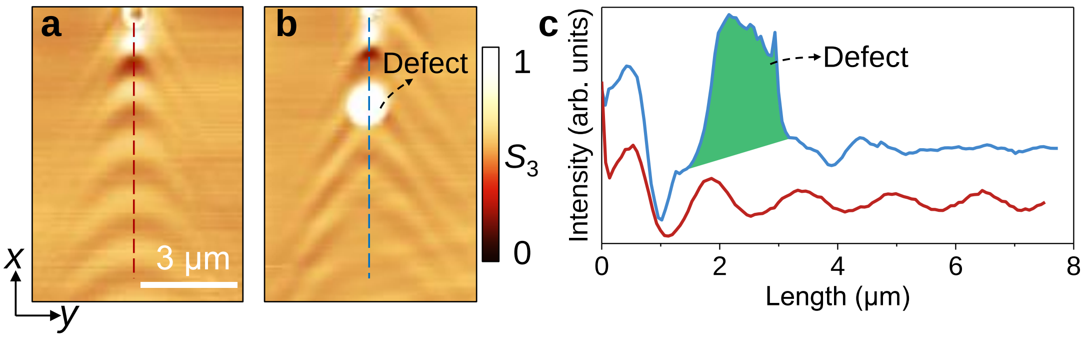

Supplementary Figure 15. Control experiments for comparison of polariton propagation with and without introducing a defect when there is no cloaking structure. (**a**, **b**) Near-field amplitude images of polariton propagation before and after introducing a defect when there is no cloaking structure. (**c**) Analysis of defect-induced polariton fringes by extracting near-field profiles from panels (a) and (b). When there are no defects, the hyperbolic wave excited by the antenna can be transmitted normally. After placing a defect in the path of the polaritons, the fringes almost disappear in the space behind the defect.

Supplementary References

1. Hu, F. et al. Imaging exciton–polariton transport in $MoSe_2$ waveguides. *Nat. Photon.* **11**, 356-360 (2017).
2. Liao, B. et al. A Multibeam Interference Model for Analyzing Complex Near-Field Images of Polaritons in 2D van der Waals Microstructures. *Adv. Funct. Mater.* **29**, 1904662 (2019).
3. Mancini, A. et al. Near-Field Retrieval of the Surface Phonon Polariton Dispersion in Free-Standing Silicon Carbide Thin Films. *ACS Photonics* **9**, 3696-3704 (2022).
4. Hu, H. et al. Doping-driven topological polaritons in graphene/α-MoO_3 heterostructures. *Nat. Nanotechnol.* **17**, 940-946 (2022).
5. Dai, S. et al. Graphene on hexagonal boron nitride as a tunable hyperbolic metamaterial. *Nat. Nanotechnol.* **10**, 682-686 (2015).
6. Wang, K. et al. Enhanced efficiency of launching hyperbolic phonon polaritons in stacked α-MoO_3 flakes. *Opt. Express* **31**, 20750-20760 (2023).
7. Zheng, Z. et al. Phonon polaritons in twisted double-layers of hyperbolic van der Waals crystals. *Nano Lett.* **20**, 5301-5308 (2020).
8. Hu, G. et al. Topological polaritons and photonic magic angles in twisted α-MoO_3 bilayers. *Nature* **582**, 209-213 (2020).
9. Duan, J. et al. Twisted Nano-Optics: Manipulating Light at the Nanoscale with Twisted Phonon Polaritonic Slabs. *Nano Lett.* **20**, 5323-5329 (2020).
10. Chen, M. et al. Configurable phonon polaritons in twisted α-MoO_3. *Nat. Mater.* **19**, 1307-1311 (2020).
